# The longitudinal risk of mortality between invasive ductal carcinoma and metaplastic breast carcinoma

**DOI:** 10.1038/s41598-020-79166-5

**Published:** 2020-12-16

**Authors:** San-Gang Wu, Shi-Ping Yang, Wen-Wen Zhang, Jun Wang, Chen-Lu Lian, Yong-Xiong Chen, Zhen-Yu He

**Affiliations:** 1 Department of Radiation Oncology, The First Affiliated Hospital of Xiamen University, Xiamen, 361003 People’s Republic of China; 2 Department of Radiation Oncology, Hainan General Hospital (Hainan Affiliated Hospital of Medical University, Haikou,, 570311 Hainan People’s Republic of China; 3 Department of Radiation Oncology, State Key Laboratory of Oncology in South China, Collaborative Innovation Center of Cancer Medicine, Sun Yat-Sen University Cancer Center, 651 Dongfeng Road East, Guangzhou, 510060 People’s Republic of China; 4 Eye Institute of Xiamen University, Fujian Provincial Key Laboratory of Ophthalmology and Visual Science, Medical College, Xiamen University, Xiamen, 361005 People’s Republic of China

**Keywords:** Breast cancer, Cancer epidemiology

## Abstract

The management of metaplastic breast carcinoma (MBC) has largely paralleled the paradigms used for invasive ductal carcinoma (IDC) in the current National Comprehensive Cancer Network guidelines of breast cancer. However, patients with IDC and MBC have been shown to have a different prognosis, and there are significant differences in risk and failure patterns after treatment. The purpose of this study was to compare breast cancer specific survival (BCSS) and hazard function between IDC and MBC. We included patients from the Surveillance, Epidemiology, and End Results program with stage I-III IDC and MBC between 2000 and 2012. Statistical analyses were including chi-square analysis, life-table methods, multivariate Cox proportional hazards models, and propensity score matching (PSM). We identified 294,719 patients; 293,199 patients with IDC and 1520 patients with MBC. Multivariate analyses showed that the MBC subtype had significantly lower BCSS than the IDC subtype before and after PSM (*p* < 0.001). There were significant differences in the hazard curve between IDC and MBC. The hazard curve for breast cancer mortality in the IDC cohort peaked at 3 years (2%), and then changed to a slowly decreasing plateau after prolonged follow up. However, the hazard curve for breast cancer mortality in the MBC cohort peaked at 2 years (7%), then declined sharply between 3 and 6 years, and changed to a low death rate after a follow-up time exceeding 6 years. Subgroup analyses revealed that the hazard curves significantly differed between IDC and MBC after stratifying by tumor stage and hormone receptor status. Our study suggests that patients with MBC should receive more effective systemic agents and intensive follow-up because of their significantly augmented risk of death compared to IDC patients.

## Introduction

Breast cancer can be classified by up to 21 distinct histological subtypes according to the cell morphology, architecture, and growth patterns in the World Health Organization classification scheme^1^. Invasive ductal carcinoma (IDC) and invasive lobular carcinoma comprise approximately 85% of all breast cancer cases, and the remaining 15% of cases are rare subtypes of breast cancer that include metaplastic breast carcinoma (MBC)^2^. MBC has long been recognized as a unique pathologic entity comprising epithelial, squamous, mesenchymal, and/or sarcomatoid components, which occur in less than 0.02–5% of all breast cancer cases^3–6^. These tumors are generally of large tumor size, node-negative, poorly differentiated, hormone receptor and human epidermal growth factor receptor-2 (HER2)-negative^7^. Several studies with limited sample size showed comparable outcomes between IDC and MBC^8,9^. However, larger cohort studies have indicated that MBC subtype have worse outcomes compared to patients with IDC^10–12^, suggesting that MBC is a more biologically aggressive pathologic entity.

Due to the rarity of this disease, there are limited data that can be used to guide treatment, surveillance, and follow-up of MBC patients. The management of MBC has largely paralleled the paradigms used for IDC in the current National Comprehensive Cancer Network (NCCN) guidelines of breast cancer^13^. However, patients with IDC and MBC have been shown to have a different prognosis, and there are significant differences in risk and failure patterns after treatment^14–16^. We believe that if all patients with a uniform follow-up program are managed according to the current guidelines, some patients may not receive prompt diagnosis of disease progress in time, which may delay salvage treatment. Therefore, it is necessary to conduct studies assessing the patterns of breast cancer deaths in MBC, in order to propose more appropriate individualized follow-up regimens. In addition, current studies do not provide accurate information regarding changes in event probability over time. In light of this, we examined breast cancer specific survival (BCSS) and hazard function between IDC and MBC patients using real-world data from the Surveillance, Epidemiology, and End Results (SEER) program.

## Methods and materials

### Patients

The SEER program of the National Cancer Institute, USA was used to include IDC and MBC patients diagnosed between 2004 and 2012. The SEER program collects information regarding patient demographics, cancer incidence, incident course of treatment, and survival data from 18 registries representing 29% of the U.S. population. We included patients pathologically diagnosed as having stage I-III IDC and MBC who were treated with breast conserving surgery or mastectomy. IDC was identified using the International Classification of Diseases for Oncology (ICD-8500), third edition. Eligible cases of MBC were identified using the morphology codes 8560, 8562, 8570–8572, 8575, and 8980–8982. We excluded those with unavailable data for race/ethnicity, tumor grade, tumor (T) stage, nodal (N) stage, estrogen receptor (ER), and progesterone receptor (PR) status. Using data from the SEER database meant that this study was exempt from the approval process of Institutional Review Boards due to anonymised patient information.

### Variables

We included the following variables for the cohort: age; American Joint Committee on Cancer (AJCC) 6th edition staging; histological subtypes, tumor grade; T stage, N stage, ER status, PR status, surgical procedures, chemotherapy, and radiotherapy. The primary outcome of this study was breast cancer-specific survival (BCSS), which was calculated as time from initial diagnosis to date of breast cancer-specific death or last follow-up.

### Statistical analysis

The chi-squared test was used to compare the differences in patient variables between those with IDC and MBC. We used a 1:1 propensity score matching (PSM) method to balance the above patient demographics, clinicopathological, and treatment characteristics in order to reduce any potential selection bias^17,18^. The life-table method was used to calculate the annual breast cancer related-death hazard rate over time. BCSS was calculated using the Kaplan–Meier method and compared using the log-rank test, which allowing for censoring at loss to follow-up or death. Multivariate Cox regression analysis using the Backward Wald Method was used to assess the independent prognostic factors related to BCSS. Sensitivity analyses focused on the surgical procedure were performed. Statistical analyses were conducted using IBM SPSS version 22.0 (IBM Corp., Armonk, NY) and Stata/SE version 14 (StataCorp, TX, USA), and a p value less than 0.05 was regarded as statistically significant.

## Results

We identified 294,719 patients including 293,199 patients with IDC and 1520 patients with MBC. The patient demographics, clinicopathological, and treatments between IDC and MBC are listed in Table 1. Patients with MBC were more likely to be older age (≥ 65 years) (*p* < 0.001), non-Hispanic Black (*p* < 0.001), have poorly/undifferentiated disease (*p* < 0.001), larger tumor size (*p* < 0.001), nodal-negative (*p* < 0.001), and hormone receptor negative disease (*p* < 0.001). In addition, more patients with MBC were treated with mastectomy (*p* < 0.001) and chemotherapy (*p* < 0.001) but less likely to be treated with radiotherapy (*p* < 0.001). There were significantly difference in patient demographics, clinicopathological, and treatments between the IDC and MBC. PSM is an ideal method to reduce the potential selection bias of retrospective studies^17,18^. Therefore, we used the PSM to balance the patient demographics, disease characteristics, and treatments between IDC and MBC. A total of 1504 pairs of patients were completely matched after PSM using the following variables: age, race/ethnicity, grade, tumor stage, nodal stage, ER/PR status, surgical procedure, chemotherapy, and radiotherapy.

**Table 1:** The patients demographic, clinicopathological, and treatment between invasive ductal carcinoma and metaplastic breast carcinoma before and after propensity score matching.

Variables	Before PSM				After PSM			
	n	IDC (%)	MBC (%)	*p*	n	IDC (%)	MBC (%)	*p*
**Age (years)**								
< 50	69,448	69,152 (23.6)	296 (19.5)	< 0.001	588	294 (19.5)	294 (19.5)	1
50–64	112,552	112,008 (38.2)	544 (35.8)		1078	539 (35.8)	539 (35.8)	
≥ 65	112,719	112,039 (38.2)	680 (44.7)		1342	671 (44.6)	671 (44.6)	
**Race/ethnicity**								
Non-Hispanic White	212,089	211,043 (72.0)	1046 (68.8)	< 0.001	2082	1041 (69.2)	1041 (69.2)	1
Non-Hispanic Black	29,726	29,500 (10.1)	226 (14.9)		446	223 (14.8)	223 (14.8)	
Hispanic (All Races)	27,865	27,722 (9.5)	143 (9.4)		276	138 (9.2)	138 (9.2)	
Other	25,039	24,934 (8.5)	105 (6.9)		204	102 (6.8)	102 (6.8)	
**Grade**								
Well differentiated	59,334	59,265 (20.2)	69 (4.5)	< 0.001	130	65 (4.3)	65 (4.3)	1
Moderately differentiated	121,815	121,606 (41.5)	209 (13.8)		412	206 (13.7)	206 (13.7)	
Poorly/undifferentiated	113,570	112,328 (38.3)	1242 (81.7)		2466	1233 (82.0)	1233 (82.0)	
**Tumor stage**								
T1	191,685	191,246 (65.2)	439 (28.9)	< 0.001	876	438 (29.1)	438 (29.1)	1
T2	84,737	83,966 (28.6)	771 (50.7)		1538	769 (51.1)	769 (51.1)	
T3	11,598	11,384 (3.9)	214 (14.1)		420	210 (14.0)	210 (14.0)	
T4	6699	6603 (2.3)	96 (6.3)		174	87 (5.8)	87 (5.8)	
**Nodal stage**								
N0	204,440	203,251 (69.3)	1189 (78.2)	< 0.001	2358	1179 (78.4)	1179 (78.4)	1
N1	65,376	65,152 (22.2)	224 (14.7)		440	220 (14.6)	220 (14.6)	
N2	16,900	16,830 (5.7)	70 (4.6)		140	70 (4.7)	70 (4.7)	
N3	8003	7966 (2.7)	37 (2.4)		70	35 (2.3)	35 (2.3)	
**TNM stage**								
I	155,325	154,924 (52.8)	401 (26.4)	< 0.001	–	–	–	
II	106,344	105,452 (36.0)	892 (58.7)		–	–	–	
III	33,050	32,823 (11.2)	227 (14.9)		–	–	–	
**ER status**								
Negative	65,652	64,402 (22.0)	1250 (82.2)	< 0.001	–	–	–	
Positive	229,067	228,797 (78.0)	270 (17.8)		–	–	–	
PR status								
Negative	96,833	95,515 (32.6)	1318 (86.7)	< 0.001	–	–	–	
Positive	197,886	197,684 (67.4)	202 (13.3)		–	–	–	
**ER/PR status**								
ER + and PR +	194,357	194,235 (66.2)	122 (8.0)	< 0.001	244	122 (8.1)	122 (8.1)	1
ER- or PR-	38,239	38,011 (13.0)	228 (15.0)		450	225 (15.0)	225 (15.0)	
ER- and PR-	62,123	60,953 (20.8)	1170 (77.0)		2314	1157 (76.9)	1157 (76.9)	
**Surgery**								
Breast conserving surgery	169,477	168,828 (57.6)	649 (42.7)	< 0.001	1292	646 (43.0)	646 (43.0)	1
Mastectomy	125,242	124,371 (42.4)	871 (57.3)		1716	858 (57.0)	858 (57.0)	
**Chemotherapy**								
No/unknown	168,269	167,654 (57.2)	615 (40.5)	< 0.001	1208	604 (40.2)	604 (40.2)	1
Yes	126,450	125,545 (42.8)	905 (59.5)		1800	900 (59.8)	900 (59.8)	
**Radiotherapy**								
No/unknown	137,677	136,869 (46.7)	808 (52.2)	< 0.001	1604	802 (53.3)	802 (53.3)	1
Yes	157,042	156,330 (53.3)	712 (46.8)		1404	702 (46.7)	702 (46.7)	

With a median follow-up of 72 months (range, 0–143 months), a total of 24,572 patients died with breast cancer-related disease, including 24,260 (8.3%) IDC and 312 (20.5%) MBC patients, respectively.

**Figure 1:**
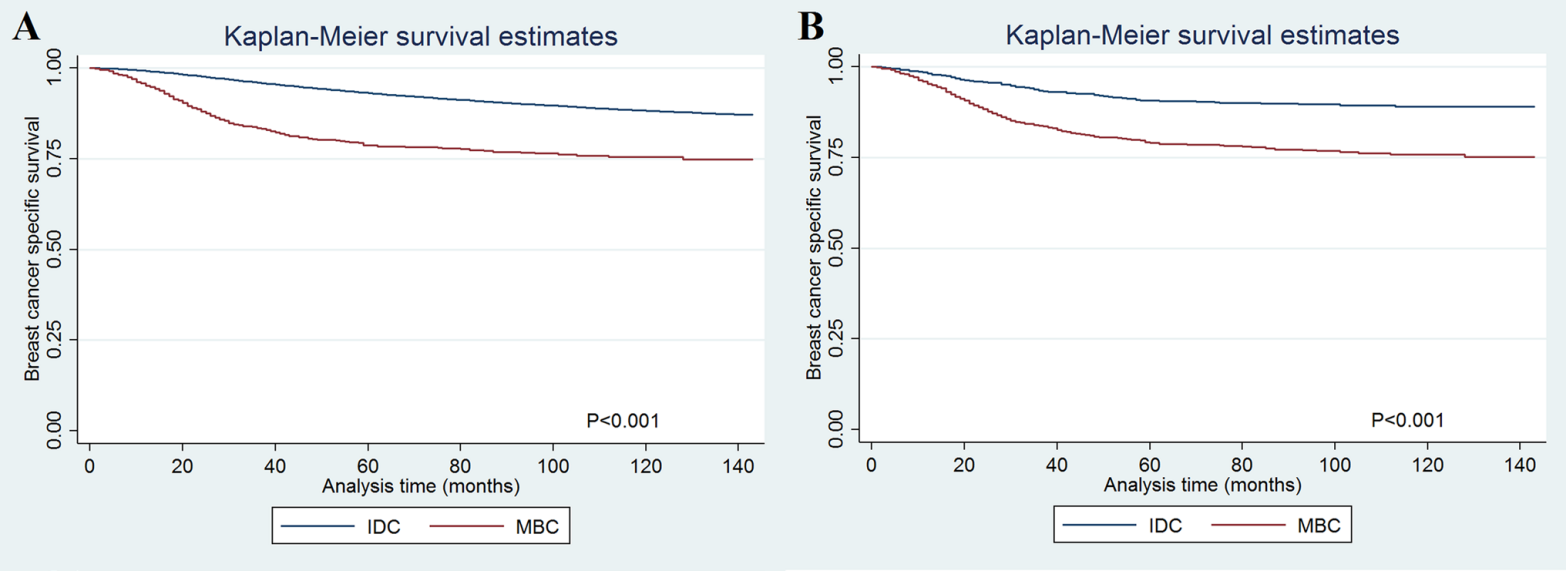
The breast cancer-specific survival between invasive ductal carcinoma and metaplastic breast carcinoma before (**A**) and after (**B**) propensity score matching.

**Figure 2:**
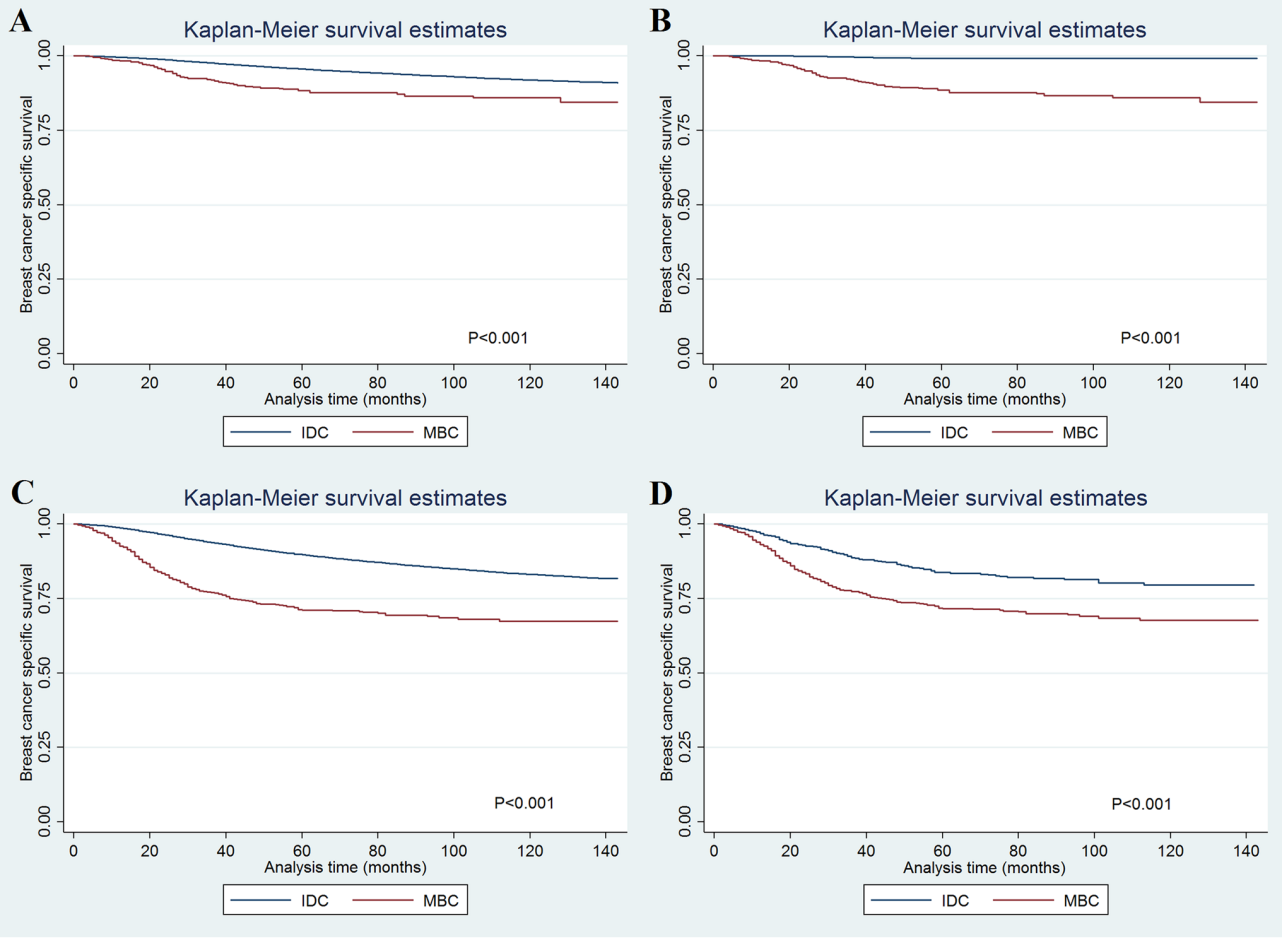
The breast cancer-specific survival between invasive ductal carcinoma and metaplastic breast carcinoma before [breast conserving surgery cohort: (**A**); mastectomy cohort: (**C**) and after (breast conserving surgery cohort: (**B**); mastectomy cohort: (**D**)] propensity score matching according to surgical procedure.

The multivariate analyses showed that histological subtype was an independent prognostic factor related to BCSS before PSM (Table 2). Patients with MBC had lower BCSS compared to IDC (hazards ratio [HR] 1.498, 95% confidence interval [CI] 1.338–1.676, *p* < 0.001). The 5-year BCSS was 93.1% and 78.6% in patients with IDC and MBC, respectively (*p* < 0.001) (Fig. 1A). After PSM, the results also indicated that those with MBC had lower BCSS than IDC patients (HR 2.708, 95%CI 2.217–3.312, *p* < 0.001) (Table 2). The 5-year BCSS was 90.7% and 79.0% in patients with IDC and MBC after PSM, respectively (Fig. 1B). Sensitivity analyses replicated similar findings after stratification by the surgical procedure (Fig. 2A–D).

**Table 2:** Multivariate analysis on prognostic factors for breast cancer specific survival.

Variables	Before PSM			After PSM		
	HR	95%CI	*p*	HR	95%CI	*p*
**Age (years)**						
< 50	1			1		
50–64	1.008	0.976–1.042	0.627	1.095	0.837–1.431	0.509
≥ 65	1.541	1.489–1.594	< 0.001	1.358	1.032–1.789	0.029
**Race/ethnicity**						
Non-hispanic white	1			1		
Non-hispanic black	1.334	1.289–1.382	< 0.001	1.159	0.904–1.485	0.245
Hispanic (all races)	0.988	0.947–1.030	0.568	0.970	0.701–1.342	0.855
Other	0.775	0.737–0.816	< 0.001	0.911	0.612–1.356	0.646
**Grade**						
Well differentiated	1			1		
Moderately differentiated	1.943	1.827–2.067	< 0.001	1.320	0.554–3.145	0.531
Poorly/undifferentiated	3.042	2.858–3.238	< 0.001	1.899	0.840–4.293	0.123
**Tumor stage**						
T1	1			1		
T2	2.101	2.036–2.168	< 0.001	2.932	2.032–4.231	< 0.001
T3	3.321	3.167–3.481	< 0.001	6.381	4.285–9.502	< 0.001
T4	4.494	4.269–4.731	< 0.001	8.308	5.355–12.890	< 0.001
**Nodal stage**						
N0	1			1		
N1	1.918	1.858–1.980	< 0.001	1.733	1.366–2.198	< 0.001
N2	3.269	3.139–3.405	< 0.001	2.280	1.651–3.150	< 0.001
N3	5.013	4.788–5.248	< 0.001	4.504	3.147–6.448	< 0.001
**ER/PR status**						
ER + and PR +	1			1		
ER− or PR−	1.581	1.524–1.641	< 0.001	0.838	0.627–1.121	0.234
ER− and PR−	1.984	1.924–2.046	< 0.001	0.769	0.541–1.092	0.142
**Surgery**						
Breast conserving surgery	1			1		
Mastectomy	1.096	1.064–1.130	< 0.001	1.818	1.388–2.382	< 0.001
**Chemotherapy**						
No/unknown	1			1		
Yes	0.832	0.806–0.858	< 0.001	0.718	0.570–0.904	0.005
**Radiotherapy**						
No/unknown	1			1		
Yes	0.741	0.720–0.763	< 0.001	0.781	0.630–0.970	0.025
**Histological subtype**						
IDC	1			1		
MBC	1.498	1.338–1.676	< 0.001	2.708	2.217–3.312	< 0.001

**Figure 3:**
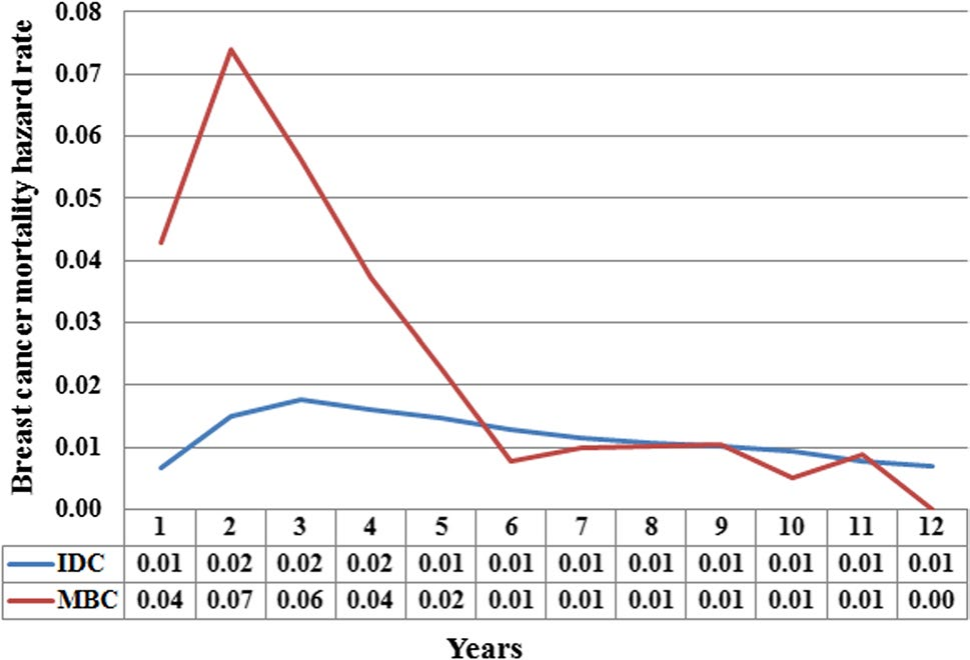
Annual hazard rates for breast cancer mortality in invasive ductal carcinoma and metaplastic breast carcinoma.

There were significant differences in the hazard curves between IDC and MBC patients (Fig. 3). The hazard curve for breast cancer mortality in the IDC cohort peaked at 3 years, and then changed to a slowly decreasing plateau after prolonged follow-up. However, the hazard curve for breast cancer mortality in the MBC cohort peaked at 2 years, then declined sharply between 3 and 6 years, and changed to a low death rate after a follow-up time exceeding 6 years.

**Figure 4:**
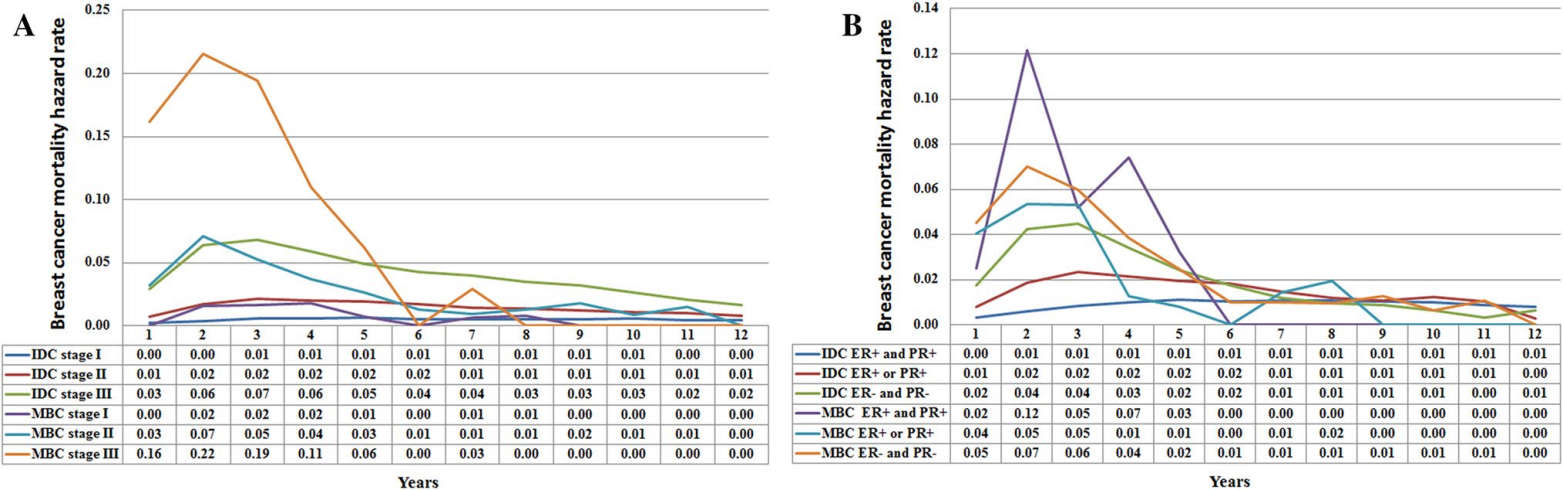
Annual hazard rates for breast cancer mortality in invasive ductal carcinoma and metaplastic breast carcinoma according to tumor stage (**A**) and hormone receptor status (**B**).

Then we conducted stratified analyses according to various tumor stage and hormone receptor status. The hazard curve showed that there were different patterns of breast cancer mortality with different tumor stage in IDC and MBC patients (Fig. 4A). The hazard function in the IDC and MBC patients were dominated by patients with advanced tumor stage. However, the hazard curve showed that MBC patients had a significantly higher risk of breast cancer mortality compared to IDC patients by various tumor stages. In MBC patients with stage III disease, the hazard curve peaked at 2 years (22.0%), and declined sharply between 3 and 6 years (19.0–0% from 3 to 6 years). In MBC patients with stage II disease, the hazard curve also peaked in 2 years (7%), and gradually decreased from 5% in 3 years then to 1% in 6 years. A low risk of breast cancer mortality was maintained after extended follow-up. However, for stage I MBC patients, there was a lower risk of breast cancer mortality (2%) in years 2–4 and a very low risk of breast cancer mortality (0–1%) after 5 years of follow-up. Similarly, for patients with stage I and II IDC, the annual risk of breast cancer mortality was extremely low (0–2%), and there was an approximate 1% risk of breast cancer mortality each year after 7-years of follow-up. For patients with stage III IDC, the peak risk of breast cancer mortality was after 3 years and then it gradually decreased. Interestingly, the risk of breast cancer mortality in stage III IDC was highest in the 6-year follow-up period and decreased year by year thereafter.

**Figure 5:**
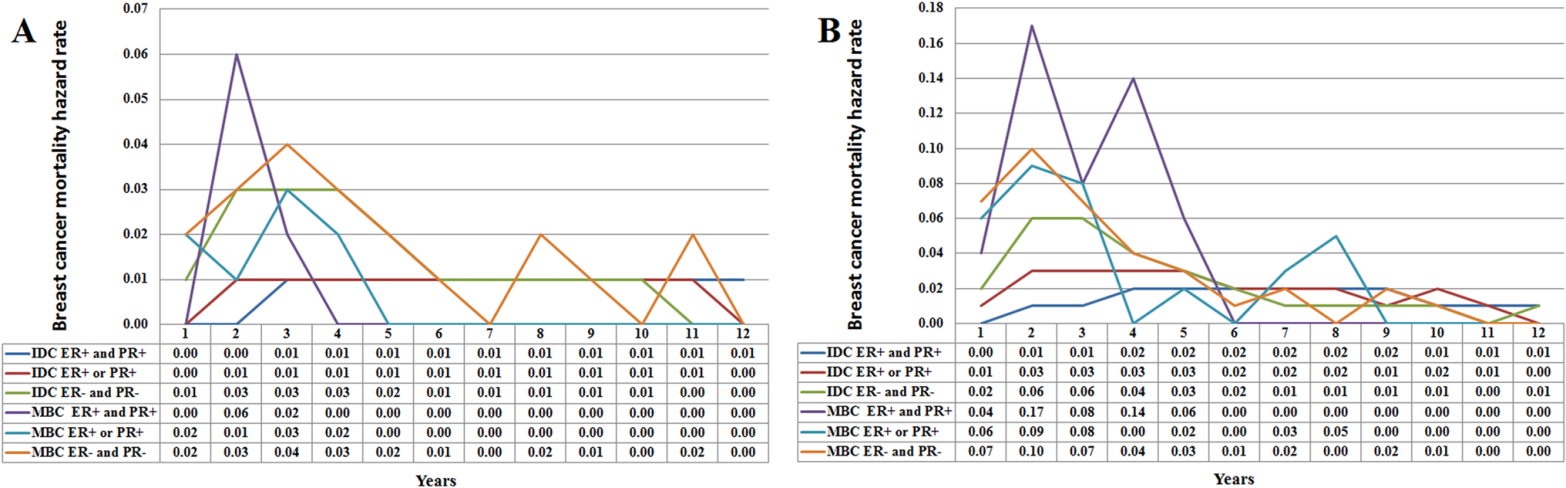
Annual hazard rates for breast cancer mortality in invasive ductal carcinoma and metaplastic breast carcinoma by hormone receptor status in breast conserving surgery cohort (**A**) and mastectomy cohort (**B**).

There were also different patterns of breast cancer mortality with different hormone receptor status in IDC and MBC patients (Fig. 4B). In IDC patients, the hazard rate was much lower and a sharp peak was not evident in patients with single or both hormone receptor positive diseases, while the hazard rate in patients with ER- and PR-negative disease peaked at 2–3 years (4%), and then declined gradually to 1% in a 7-year period. However, in MBC patients with ER- and PR-positive disease, the hazard curve had two peaks. The first peak occurred at 2 years (12%), and the second peak between 4 years (7%). In addition, the hazard curve for breast cancer mortality in single (5%) or both hormone receptor-negative diseases (6–7%) both peaked at 2–3 years. Interestingly, after 6 years of follow-up, the overall risk of breast cancer-specific death had decreased in the three MBC subgroups, and the hazard curve across the six subgroups, suggesting that the risk of breast cancer mortality was similar and tended to exhibit a very low hazard rate between the two histological subtypes. Similar results were found in patients who received breast conserving surgery (Fig. 5A) or mastectomy cohort (Fig. 5B).

## Discussion

In this study, we assessed the change in the risk of breast cancer mortality over time between IDC and MBC subtypes and found significantly different patterns of hazard function between the two histological subtypes.

Our study largely supports previous studies that MBC patients were more likely to be older and of black ethnicity. Furthermore, they tended to have larger tumor size, higher tumor grade, less nodal involvement, and more hormone receptor-negative tumors compared to IDC^6,19^. In addition, more patients had received mastectomy and chemotherapy, which may be related to larger tumor size and highly aggressive biological behavior. The aggressive prognostic factors were detected more frequently in MBC. However, MBC presents a lower risk of nodal involvement despite larger tumor size. In addition, patients with MBC have a higher risk of hematogenous spread, while more regional lymphatic spread was observed in IDC patients^7,20^. Therefore, there appear to be somewhat different tumor proliferation mechanisms between MBC and typical ductal origin tumors.

Whether the survival outcomes of MBC patients are indeed lower than for IDC patients remain to be fully delineated. A previous study by Barquet-Muñoz et al*.* showed comparable survival outcomes in MBC patients compared to other histological subtypes including IDC and invasive lobular carcinoma with unfavorable immunohistochemical factors^8^. Another study from Korea also showed that there was no significant difference in survival outcomes between IDC and MBC patients^9^. However, there were only 24 and 29 patients in the MBC groups in the aforementioned two studies respectively, which limited representation of the study participants to the entire population of MBC. In recent years, several population-based studies have indicated that there appears to be lower disease-specific survival in MBC patients compared to IDC patients^10–12^. In our study, we used a large cohort to compare the outcomes between MBC and IDC diagnosis, and our results are consistent with the findings from previous population-based studies^10–12^. Indeed, we show that even after accounting for the risk factors through PSM, lower BCSS in MBC patients still persist.

To the best of our knowledge, our study was the largest cohort to compare hazard function between IDC and MBC patients. In our study, we observed significant differences in the hazard function between the two histological subtypes. The MBC deaths peaked at 2 years (7%) and decreased quickly thereafter, while the hazard curve peaked at 3 years in IDC (2%) and continued to fall over time after prolonged follow-up. The hazard function in IDC patients was similar to previous studies^21,22^. However, the hazard function was comparable between IDC and MBC after 6-years of diagnosis and follow-up. Our results suggested that the biologic mechanisms responsible for early and late breast cancer mortality rates are fundamentally different. When stratified by tumor, we found that breast cancer death differences between IDC and MBC patients were greatest in patients with stage II-III diseases. Our study suggests that poor prognosis in MBC may be associated with early tumor recurrence and decreased therapeutic efficacy of the current systemic management of this patient subset. Use of adjuvant systemic chemotherapy in accordance with the current NCCN breast cancer guidelines are based on clinical tumor stage rather than on histologic subtype^13^. Although the rate of adjuvant chemotherapy receipt in MBC patients was higher than for IDC patients, the clinical response to systemic chemotherapy in MBC was significantly poorer than IDC. The partial tumor response in patients with metastatic MBC was 6–21%, while 18–21% had stable disease, and approximately 80% experienced disease progress after palliative systemic chemotherapy^14,23,24^. Therefore, it is necessary to further explore the optimal management of MBC.

Hormone receptor status has been shown to be one of the main indicators for prognosis in IDC. Our study also confirmed that hormone receptor-positive IDC patients have a significantly lower risk of breast cancer death than hormone receptor-negative patients, and hormone receptor-positive patients had a stable risk of breast cancer death over a 1–12 year period (0–1%). However, the hazard curve in hormone receptor-negative patients peaked 2–3 years after diagnosis (4%), and the hazard curve crossed with hormone receptor-positive patients after 7-years of follow-up, which was similar to previous studies^21,22^. In patients with MBC, most of the current studies have suggested that there is no significant correlation between hormone receptor status and MBC survival^19,25^. Our study found that for patients with MBC, the effects of hormone receptor status on the risk of breast cancer death were significantly different. The peaks in breast cancer death in different hormone receptor states all occurred over 2 years but the risk of breast cancer death in hormone receptor-positive disease was significantly higher than that of single or both hormone receptor-negative disease (12% vs. 5–7%). Furthermore, the risk of breast cancer death among the three groups was extremely low after 6-years of follow-up and the hazard curves did cross. Given that current practices in treatment and follow-up strategies for MBC do not consider the unique biology of MBC, these typically follow the paradigms used for patients with IDC^13^. Based on our findings, patients with MBC should receive more effective systemic agents and intensive follow-up because of their significantly augmented risk of death compared to IDC patients.

Additional to the difference in chemosensitivity between MBC and IDC, molecular factors may also be the main driver of aggressive MBC disease. The potential for epithelial-to-mesenchymal transition and cancer stem cell characteristics with self-renewal capabilities of MBC tumors seem to be potential mechanisms that drive resistance to circumvent traditional treatments and result in a tendency to metastasize^26^. Therefore, therapies that target epithelial-to-mesenchymal transition and cancer stem cells may lead to better outcomes for patients with MBC. In addition, several potentially targetable gene mutations also present in MBC and have demonstrated favorable clinical responses to corresponding targeted therapies^26,27^. However, there have been limited approaches, which demonstrate an overwhelming benefit from targeted therapy in current clinical practice^28^. Recent studies have shown frequent overexpression of programmed death-ligand 1 (PD-L1) in MBC^27,29^. Investigations of such novel management combined with conventional chemotherapy are required in MBC, which is associated with a poor outcome and a lack of effective therapeutic strategies.

Our study has certain limitations. The first limitation of this study is the inherent bias in any retrospective analyses. Second, there is a lack of central pathologic review in the SEER program. Third, the sparse coding of HER2 status before 2009 in the SEER database limited the analysis of our study with respect to the effect of HER2 status on hazard function. However, a previous study has shown that MBC patients with HER2-positive disease have better outcomes, suggesting that HER2-positive MBC may be responsive to HER2-directed therapy^19^. In addition, the lack of data on locoregional recurrence and distant metastasis in the SEER program limited evaluation of the effects of both local and systemic treatments. Finally, the under-reporting of chemotherapy treatment of patients, and the specific agents and number of cycles of chemotherapy were also not mentioned in the SEER database. However, the primary strength of our study was that this was the largest cohort study to compare the hazard function of MBC and IDC patients, which has important guiding indications for follow-up strategies of the two histological subtypes.

In conclusion, our study suggests that MBC is a biologically different tumor from IDC, and MBC is also associated with poorer outcome than IDC. In addition, the patterns of hazard rates of breast cancer-related death significantly differed between IDC and MBC. More studies are needed to develop more effective systemic management of MBC and intensive follow-up should be performed in MBC patients, in particular.
